# The Role of a Newly Synthesized Antimicrobial Peptide (KK)_2_-KWWW-NH_2_ in Modulating Phosphatidylinositol Monolayer Properties in the Presence of Ascorbic Acid

**DOI:** 10.3390/ijms262110344

**Published:** 2025-10-23

**Authors:** Iwona Golonka, Aleksandra Sebastiańczyk, Izabela W. Łukasiewicz, Katarzyna E. Greber, Wiesław Sawicki, Witold Musiał

**Affiliations:** 1 Department of Physical Chemistry and Biophysics, Wroclaw Medical University, Borowska 211A, 50-556 Wrocław, Poland; iwona.golonka@umed.wroc.pl (I.G.); aleksandra.sebastianczyk@student.umw.edu.pl (A.S.); izalukasiewicz@gmail.com (I.W.Ł.); 2 Department of Physical Chemistry, Faculty of Pharmacy, Medical University of Gdańsk, Al. Gen. J. Hallera 107, 80-416 Gdańsk, Poland; katarzyna.greber@gumed.edu.pl (K.E.G.); wieslaw.sawicki@gumed.edu.pl (W.S.)

**Keywords:** Langmuir isotherm, compression reversibility factor, antibacterial peptides, ascorbic acid, 3-O-ethyl-L-ascorbic acid, phosphatidylinositol membrane

## Abstract

Ascorbic acid (AA) and its derivatives (EAA), due to their antioxidant properties, may offer potential support in acne therapy. The aim of this study was to evaluate the effect of compound P6—(KK)_2_-KWWW-NH_2_—in the presence of AA or EAA on the stability and organization of phosphatidylinositol (PI) monolayers. The conducted experiments showed that the monolayers were in the expanded liquid state (37.45–48.35 mN/m) or in the transitional phase between the expanded liquid and condensed states (51.06–56.82 mN/m). Compression and decompression isotherms indicated the highest flexibility for the PI + P6 system, with the compression reversibility coefficient (*R*_v_) ranging from 87.34% to 97.77%, increasing with temperature in successive loops. The surface pressure vs. time dependence after compound injection into the subphase revealed a decrease in monolayer surface pressure followed by stabilization after approximately 300 s for the PI + P6 + AA and PI + P6 + EAA systems. In contrast, for the PI + P6 system at 35 °C, an increase in surface pressure was observed.

## 1. Introduction

Excessive and inappropriate use of antibiotics has led to a significant decrease in their effectiveness and an increase in the number of resistant microorganisms [1]. One of the promising solutions in the fight against bacterial resistance are antimicrobial peptides (AMPs), which are characterized by a broad spectrum of action and the ability to destroy both Gram-positive and Gram-negative bacteria [2,3]. Due to their positive charge, amphipathic nature, and hydrophobicity, AMPs interact with microbial cell membranes, leading to their destabilization and lysis [4,5,6]. This mechanism of action limits the possibility of resistance development, which is a major advantage over traditional antibiotics. In comparison, organocyclophosphazenes, belonging to the group of small-molecule antimicrobial agents, exhibit strong activity against bacteria and fungi such as *Mycobacterium tuberculosis*, *Bacillus cereus*, *Klebsiella pneumoniae*, and *Candida* spp., as well as cytotoxic properties against cancer cells [7]. These compounds can also serve as carriers of biologically active substances and components of biomedical materials; however, their potential toxicity and limited chemical stability pose challenges for clinical applications. Silver nanoparticles (AgNPs) represent another class of compounds with a broad spectrum of antibacterial and antifungal activity. They have been shown to act mainly through the generation of reactive oxygen species and disruption of microbial cell membranes [8]. Despite their high efficacy, concerns regarding their biocompatibility and potential environmental impact limit their widespread use.

Peptide P6—(KK)_2_-KWWW-NH_2_—has been developed as a potential agent for treating skin infections, including acne vulgaris. Its structure, combining polar lysine and nonpolar tryptophan residues, ensures a balanced hydrophilic–hydrophobic profile. P6 shows strong antioxidant and antibacterial activity, with MIC and MBC values of 250 mg/L against *S. aureus*, and inhibits *C. acnes* growth (1 mm zone) when released from bacterial cellulose (BC) carriers. It is non-cytotoxic to L929 fibroblasts, confirming its safety [9]. Analysis of the results obtained by the Langmuir method showed that P6 formed a stable monolayer, suggesting its potential to form a continuous film on the skin surface, enhancing its anti-acne effect. Thermogravimetric analysis confirmed that the P6 peptide in its powder form is thermally stable, while molecular dynamics simulations indicated its deep localization within the water layer and strong hydrophilic nature (D = 0.2763 × 10^−5^ cm^2^/s) [10]. With moderate lipophilicity (logP ≈ 0), comparable to camphor—a known skin penetration enhancer—P6 demonstrates favorable skin permeation properties. Its interactions with model lipid membranes (azolectin, lecithin) suggest promising potential for further antimicrobial studies [11,12]. Moreover, since *P. acnes* strains contain phosphatidylinositol, along with DPG and PG, as one of their major phospholipids, further investigations were undertaken to assess the effect of P6 on the phosphatidylinositol monolayer [13].

In dermatology, increasing attention is also paid to vitamin C, known for its anti-inflammatory and antioxidant effects, as well as for the reduction in hyperpigmentation. The chemically unstable form of ascorbic acid (AA) is sometimes replaced by ethyl ascorbic acid (EAA), which is more stable and better at penetrating the skin barrier [14]. Combining peptides such as P6 with vitamin C derivatives may constitute a modern therapeutic approach for the treatment of inflammatory skin conditions, such as acne vulgaris, which affects up to 30% of people aged 11–30 and may lead to significant psychological consequences [15]. Previous studies on phosphatidylinositol monolayers have shown that the presence of ascorbic acid significantly alters the physicochemical properties of the lipid film [16].

Human skin temperature reflects heat exchange at the body–environment boundary and varies by facial region. The highest temperature is observed on the forehead, while the lowest occurs on the ears or cheeks. Bilateral symmetry of temperatures on cheeks and ears was consistently observed, with a difference between the sides not exceeding 0.3–0.5 °C [17,18].

The aim of this study was to investigate how the antimicrobial peptide P6—the most hydrophilic among the peptides examined by the authors—interacts with phosphatidylinositol (PI) monolayers, a key phospholipid present in *P. acnes*, and to compare its behavior with that observed for lecithin, azolectin, and other peptides previously studied. Considering the variability of skin surface temperature, Langmuir monolayer experiments were conducted within the 20–35 °C range to closely mimic physiological skin conditions. Furthermore, the combined effects of P6 with ascorbic acid and its ethylated derivative on the organization, compressibility, hysteresis, and surface pressure dynamics of phosphatidylinositol monolayers were evaluated, which may reveal potential synergistic interactions and new possibilities for enhancing anti-acne efficacy.

## 2. Results

### 2.1. Compression Isotherms of the PI Monolayer on an Aqueous Subphase Containing Peptide and AA or EAA

A phosphatidylinositol monolayer was formed on two types of subphases containing different combinations of peptide P6, P6 with either AA or its derivative EAA (Table 1). Compression isotherms were subsequently recorded.

**Figure 1:**
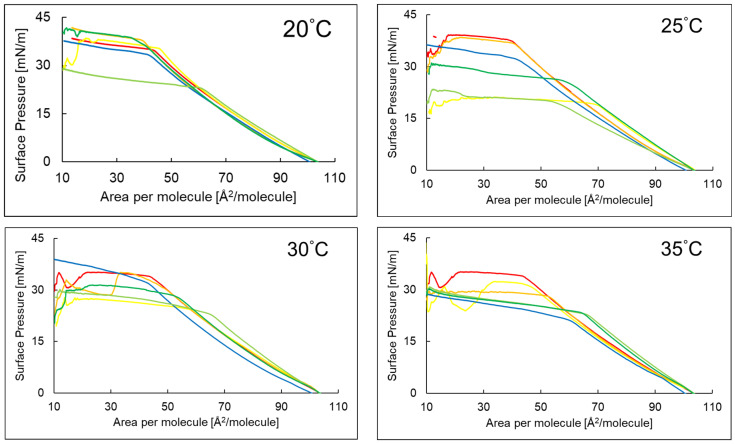
The course of the compression isotherms of the systems PI (**—**), PI + AA (**—**), PI + EAA (**—**), PI + P6 (**—**), PI + P6 + AA (**—**), PI + P6 + EAA (**—**) at temperatures of 20–35 °C.

The compression isotherms of the PI monolayer on the aqueous subphase containing AA and its derivative EAA were published in the previous work [16]. In the present study, these isotherms were referred to (Figure 1) as reference samples, which enabled the assessment of changes in the compression curves after adding the P6 peptide to the subphase.

The compression isotherm of the PI + P6 system, compared to the other systems studied, was shifted toward smaller surface areas per molecule. However, when AA or EAA was also added to the PI + P6 system, the isotherm shifted toward larger molecular areas compared to the PI + P6 system. The increase in surface pressure above zero for the phosphatidylinositol monolayer over subphase with EAA and AA at the four temperatures studied occurs at an average surface area per molecule of 104.40 Å^2^/molecule. Comparing the isotherms of the PI monolayers with P6 and EAA, and the PI monolayers with P6 and AA in the aqueous subphase, it can be seen that the PI monolayers with P6 and EAA exhibited collapse at higher surface pressures at temperatures of 20 °C, 25 °C, and 30 °C than the PI monolayers with P6 and AA. However, at 35 °C, the moment of collapse and the isotherms was similar for both systems: PI with P6 and EAA and PI with P6 and AA.

Based on the π–A isotherm diagrams, key parameters characterizing the Langmuir monolayers formed by the tested layer-forming compounds were determined (Table 2). These include: A_lift-off_—the surface area per molecule at which the surface pressure (π) began to rise above 0 mN/m; π_collapse_—the surface pressure at which the monolayer collapses; A_collapse_—the surface area per molecule at the collapse point, χ (A_collapse_/A_lift-off_); a_LE/LC_—the slope of the linear region of the π–A isotherm, corresponding to the dynamic increase in surface pressure.

The A_lift-off_ and A_collapse_ parameters for the monolayer with P6 in the subphase at all temperatures showed the lowest value. The ratio of collapse area to lift-off area for PI + P6 and PI + P6 + EAA decreased with increasing temperature.

### 2.2. Compressibility of the PI Monolayer on Aqueous Subphase with Peptide, AA, or EAA

**Figure 2:**
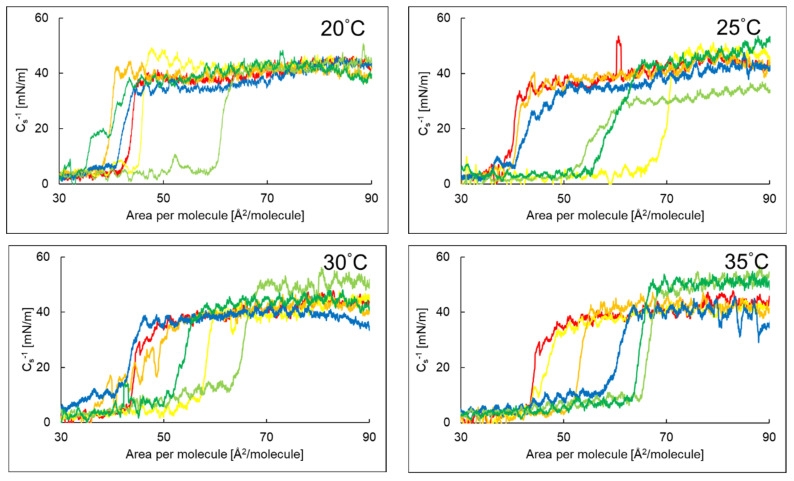
Dependence of the compressibility coefficient depending on the surface area per molecule of the systems PI (**—**), PI + AA (**—**), PI + EAA (**—**), PI + P6 (**—**), PI + P6 + AA (**—**), PI + P6 + EAA (**—**) at temperatures of 20–35 °C.

Figure 2 presents the dependence of the compressibility coefficient of the phosphatidylinositol monolayer on the surface area per molecule in the presence of AA, EAA, or the antimicrobial peptide P6 in the aqueous subphase at temperatures of 20 °C, 25 °C, 30 °C, and 35 °C.

Table 3 summarizes the maximum values of the compressibility coefficient, indicating that PI monolayers with EAA and P6 in the aqueous subphase at 25 °C and 35 °C, as well as PI with AA and P6 at 20 °C, 30 °C and 35 °C, existed in the expanded liquid state. The remaining monolayers were found to be in a transition state between the expanded liquid phase and the condensed liquid phase.

The value of the compressibility coefficient varies depending on the system under investigation and the measurement temperature. In the case of a monolayer on an aqueous subphase, it increased slightly with rising temperature from 46.23 to 49.95 mN/m in the range of 20–35 °C. Adding P6 to the aqueous subphase caused a decrease in the *C_S_*^−1^ value of the monolayer at temperatures of 25, 30, and 35 °C. The PI + P6 + EAA system demonstrated high compressibility while maintaining cohesion over a wide temperature range.

### 2.3. Compression and Expansion of the PI Monolayer on an Aqueous Subphase Containing Peptide, AA, or EAA

**Figure 3:**
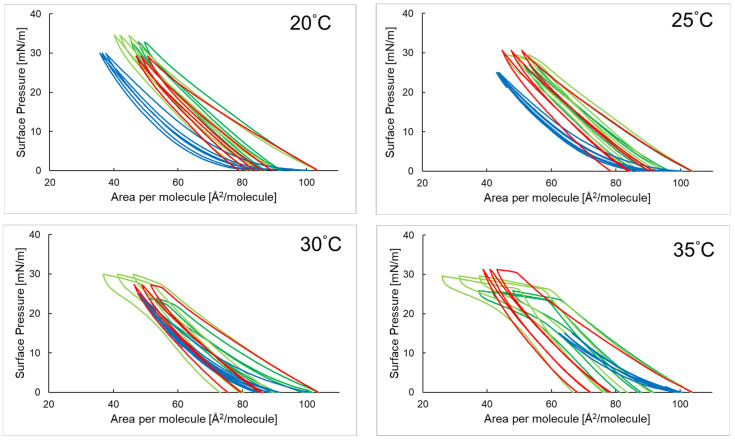
Hysteresis course of phosphatidylinositol PI monolayer (**—**) [16] in the presence of P6; PI + P6 (**—**), ascorbic acid PI + P6 + AA (**—**) and 3-O-ethyl-ascorbic acid PI + P6 + EAA (**—**) in the aqueous subphase at temperatures of 20–35 °C.

Figure 3 shows three hysteresis loops representing the compression and decompression isotherms of a phosphatidylinositol monolayer with 3-O-ethyl ascorbic acid and ascorbic acid in the presence of the antimicrobial peptide P6 at temperatures of 20 °C, 25 °C, 30 °C, and 35 °C.

In the temperature range of 20–35 °C, the shapes of the hysteresis loops for the PI + P6 system are similar—each subsequent loop run close to the previous one, and the compression and decompression paths nearly overlapped. As temperature increased, a decrease in surface pressure was observed at the maximum compression point. The hysteresis curves for the PI + P6 + EAA and PI + P6 + AA systems follow a similar pattern, with the width of subsequent loops decreasing slightly. At temperatures of 30 °C and 35 °C, a flattening of the compression lines was visible, and the loops themselves occurred over a wider range of area per molecule (area per molecule) than in the case of the PI + P6 monolayer. In most cases, for the P6 systems, an increase in the *R*_v_ (%) parameter value was observed with each subsequent loop, regardless of temperature (as shown in Table 4). A similar phenomenon also occurred for the PI + P6 + AA system. The exception was the PI + P6 + EAA system, which did not show this relationship.

### 2.4. Time-Dependent Surface Pressure of Evaluated Monolayers in the Studied Systems

**Figure 4:**
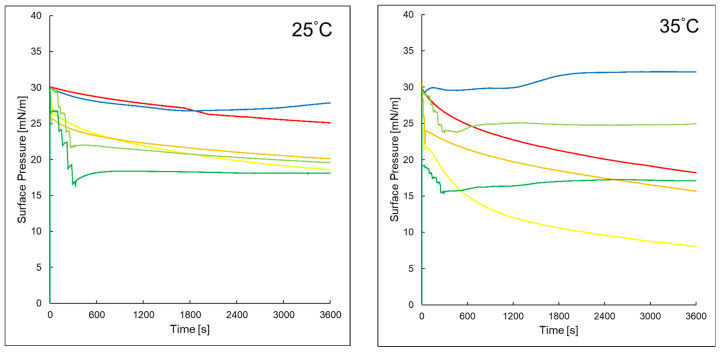
Surface pressure values of assessed layers versus time at temperatures of 25 °C and 35 °C of the tested systems: PI (**—**), PI + AA (**—**), PI + EAA (**—**), PI + P6 (**—**), PI + P6 + AA (**—**), PI + P6 + EAA (**—**).

Figure 4 presents the changes in surface pressure over time for the studied systems at 25 °C and 35 °C. For the pure PI monolayer, a gradual decrease in surface pressure was observed over time. At 25 °C, the surface pressure dropped from approximately 30 mN/m to about 25 mN/m. At 35 °C, the decrease is more rapid, reaching ~18 mN/m. The addition of AA to the subphase lead to a more pronounced reduction in surface pressure at both temperatures, when compared to the effect of EAA, which caused a smaller decrease. In the PI + P6 system, the surface pressure initially decreased and then increased over time at both 25 °C and 35 °C, with a more pronounced effect observed at the higher temperature. This suggested a different interaction mechanism compared to systems containing PI alone. A similar trend was observed for the PI + P6 + EAA system, where the surface pressure stabilized at approximately 18–20 mN/m, regardless of temperature.

## 3. Discussion

Cells could actively modify membrane lipid composition to maintain optimal physical properties by regulating membrane size and shape during growth. Changes in the fatty acid profile, such as the introduction of odd-numbered, branched chains (e.g., in *Staphylococcus aureus*), enabled regulation of viscosity and phase transition temperature. Acyl chain length influenced membrane thickness and physical state. The order and disorder parameters of the membrane are also influenced by temperature, hydration, pressure, pH and ionic strength. Cells responded to membrane destabilization by altering lipid composition and distribution—both lateral and transverse [19,20]. These processes significantly hindered effective treatment of infections, contributing to the growing phenomenon of antibiotic resistance. It is therefore crucial to search for new molecules that not only inhibited basic bacterial metabolic processes but also disrupted the integrity and function of cell membranes.

**Figure 5:**
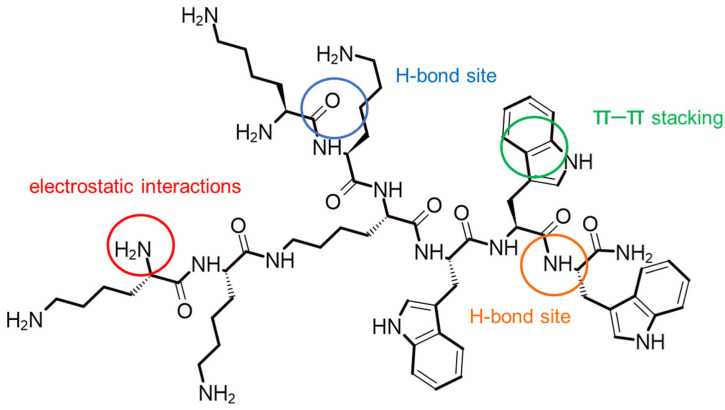
Structure of peptide P6 with possible interaction sites.

The analysis of the π–A isotherms for the PI + P6 as well as PI + P6 + AA and PI + P6 + EAA systems provided interesting information on the molecular interactions and surface properties of the studied monolayers. The shift in the PI + P6 system isotherm toward smaller surface areas per molecule may indicate a more compact organization of molecules in the monolayer, which may suggest stronger intermolecular interactions (e.g., electrostatic, hydrogen) [21]. According to available bibliography the molecules may enter into favorable interactions—Figure 5 (п-п stacking) [22,23]. The structure of P6—(KK)_2_-KWWW-NH_2_—with highlighted sites of possible interactions is shown in Figure 5.

Lysine-derived amino groups could form hydrogen bonds. Tryptophan, which had an aromatic system, could form π–π interactions, e.g., with other aromatic rings, and hydrophobic interactions, important for binding to biological membranes. It could also participate in hydrogen bonds via the NH group in the indole ring. The terminal amine group (NH_2_) could act as a hydrogen bond donor [24].

Another cause of the observed changes in the π–A isotherm could have been a change in the orientation of the molecules in the monolayer, which would have aligned more vertically with respect to the surface, or partial desorption of the PI molecules into the aqueous subphase due to the presence of the highly hydrophilic P6 compound [25,26]. Adding AA or EAA to this system shifted the isotherm toward larger molecular surfaces. This suggested that these acids introduced additional spatial elements or weakened the interactions between PI and P6 molecules, resulting in a more dispersed layer. This change may have resulted from the formation of new interactions. AA and EAA could have weakened the original PI-P6 interactions or even incorporated into the monolayer [27].

**Figure 6:**
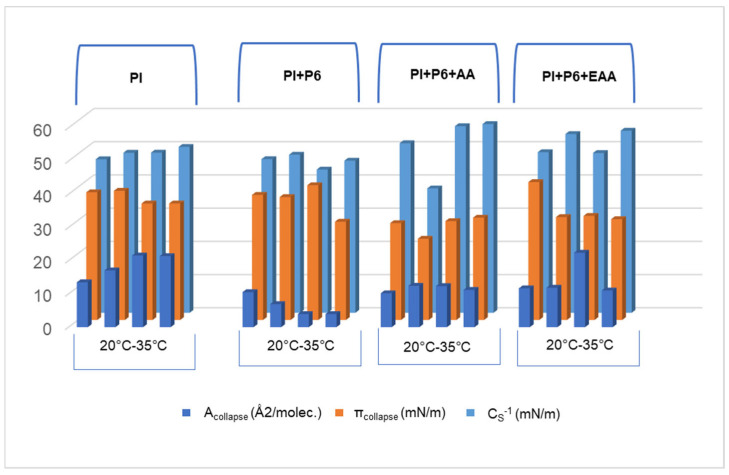
Parameter values: A_collapse_ (Å2/molec.), π_collapse_ (mN/m), *C_S_*^−1^ of the tested systems at temperature of 20–35 °C.

The addition of P6 to the subphase caused a decrease in the A_collapse_ parameter value of the PI monolayer (Figure 6). This meant that the monolayer collapses with a smaller surface area, accompanied by a slight decrease in surface pressure and a nearly stable compressibility coefficient with increasing temperature. This may have indicated a decrease in monolayer stability, but without a significant effect on the phase type it was located in. P6 likely did not interact with the hydrophobic tails, but it does influenced the organization of the PI polar heads. Adding AA or EAA to a subphase already containing P6 caused an increase in the compressibility coefficient of the PI monolayer in most cases. A_collapse_ and π_collapse_ remained at levels similar to those in the previous combinations, indicating partial compensation of the P6 effects of AA or EAA. The highest values of the compressibility coefficient are presented by the monolayer of the PI + P6 + AA system at temperatures of 30 and 35 °C, which was, as the only one, in the condensed liquid phase.

For the PI + P6 system, in the temperature range of 20–35 °C, the *R*_v_ parameter value for all three compression–decompression loops was close to 100%, indicating that the layer compression process was fully reversible. The monolayer retained its elasticity, and its stability increased after the addition of P6 to the subphase, as seen in the surface pressure vs. time graph (Figure 4) [28]. Temperature also had a significant impact on the behavior of monolayers in terms of phase transitions. As shown in Figure 3, a plateau was observed in the compression curves for the PI + P6 + AA and PI + P6 + EAA systems, indicating the occurrence of a phase transition. Importantly, at higher temperatures, this plateau shifted toward higher surface area per particle, further confirming the effect of temperature on monolayer organization [29,30].

In summary, AA in the presence of P6 has a more active effect on the monolayer by lowering surface tension and increasing the compressibility coefficient, which may facilitate penetration into the upper layers of the skin or into the acne bacterial membrane. However, it is less stable and may oxidize more quickly [31,32].

EAA typically has better penetration through the skin’s lipid barrier and greater chemical stability. This means that in pharmaceutical formulations it may last longer and reach infected areas more effectively, even though it has a weaker effect on surface tension in monolayer studies [33].

The addition of ascorbic acid to the aqueous subphase causes a reduction in the surface tension of the phosphatidylinositol (PI) monolayer. This decrease indicates strong interactions between ascorbic acid and PI molecules, leading to a reduction in surface energy [34,35]. Simultaneously, an increase in the compressibility of the monolayer was observed, indicating greater flexibility and loosening of the lipid structure, possibly due to peptide incorporation. This effect is consistent with reports showing that peptides modulate the physicochemical properties of membranes through interactions with the hydrophilic head groups of lipids. When peptide P6 is injected into the subphase containing AA and PI, a more pronounced initial drop in surface tension occurs compared to the AA + PI system alone. This is followed by a slight increase and subsequent stabilization of surface tension at a value lower than the original, suggesting that P6 initially disrupts the monolayer packing more strongly, but then reorganizes or incorporates into the lipid part of PI, leading to a new equilibrium state with enhanced stability [36].

Due to its ability to penetrate model lipid membranes and its high diffusion coefficient, P6 can be applied topically, especially when delivered via BC carriers.

## 4. Materials and Methods

### 4.1. Synthesis and Characterization of the Peptide

The synthesis, purification, and structural determination of peptide P6 were carried out as described in our earlier publication [9]. The purity and identity of the peptide were confirmed by high-performance liquid chromatography HPLC (Nexera, Shimadzu, Tokyo, Japan) and mass spectroscopy MS (Christ, Hannover, Germany) [9].

### 4.2. Langmuir Films

To investigate monolayers of phosphatidylinositol (PI, C_47_H_82_NaO_13_P, Sigma-Aldrich, St. Louis, MO, USA) on an aqueous subphase in the presence of peptide P6 and either ascorbic acid (AA, Fagron, Modlniczka, Poland) or ethyl ascorbic acid (EAA, Merck, Darmstadt, Germany), a Langmuir–Wilhelmy trough (Microtrough X, Kibron, Helsinki, Finland) was used, controlled by Filmware X 4.0 software.

The trough consisted of a Teflon tray (23.7 × 7.9 cm), two movable Teflon barriers, and a platinum wire (0.5 mm diameter, 48.2 mg) used instead of a Wilhelmy plate, ensuring a negligible contact angle. To avoid contamination, the platinum wire was cleaned with methanol and water, then flamed before each use. Barriers moved at a constant speed of 10 mm/min.

Surface cleanliness was verified by ensuring that surface tension variations did not exceed 0.30 mN/m during barrier movement. If exceeded, the cleaning procedure was repeated. All experiments were conducted in triplicate on anti-vibration tables, with temperature controlled at 20 °C, 25 °C, 30 °C, and 35 °C using a Kruss thermostat (Hamburg, Germany).

After checking the purity of the subphase, chloroform (Merck, Darmstadt, Germany) and methanol (Merck, Darmstadt, Germany) were mixed in a 9:1 volume ratio. Phosphatidylinositol was dissolved in the prepared mixture to obtain a concentration of 1.18 × 10^−6^ mol/L. The solution was applied to the surface of the aqueous subphase. After complete evaporation of the solvent, the monolayer was symmetrically compressed to a surface pressure of 5 mN/m. Then, 30 μL of an aqueous solution of the tested peptide with a concentration of 1.27 × 10 − 3 mol/L and 30 μL of an AA or EAA solution with a concentration of 1.27 × 10 − 3 mol/L were introduced into the subphase. After 15 min of system stabilization, the barriers were moved inward at a speed of 10.02 mm/min. The obtained results were processed using Microsoft Excel calculation sheets.

### 4.3. Hysteresis Measurements

Compression–decompression isotherms were recorded at a constant barrier speed of 10 mm/min. Each sample underwent three full cycles. The reversibility factor (*R*_v_) was calculated based on the method by Georgiev et al. [37]:

(1) $$Rv=\frac{{(\int_{A_{collapse}}^{A_{lift\;off}}\pi dA)}_{expansion}}{{(\int_{A_{collapse}}^{A_{lift\;off}}\pi dA)}_{compression}}\;\cdot 10$$

where *R_v_*—compression reversibility factor, *A_lift−off_*—surface area at lift-off, *A_collapse_*—surface area at collapse

### 4.4. Compressibility Modulus of the Monolayer

The compressibility modulus *C_S_*^−1^, indicating the monolayer’s resistance to compression, was calculated from the *π-A* isotherms:

(2) $$C_{S}^{-1}=-A\left(\frac{d\pi }{dA}\right)$$

where *C_S_*^−1^—compressibility factor [mN/m], *A*—surface area per molecule [Å^2^/molecule], *π*—surface pressure [mN/m].

### 4.5. Surface Pressure Changes over Time

To monitor the time-dependent surface pressure response, 15 μL of PI solution was applied to the subphase and allowed to evaporate for 15 min. The monolayer was compressed to 30 mN/m, approximating the conditions of biological membranes.

Subsequently, 30 μL of peptide P6 and 30 μL of ascorbic acid or ethyl ascorbic acid (both at 1.27 × 10^−3^ mol/L) were introduced into the subphase. Surface pressure was measured over 60 min at constant surface area, at two temperatures: 25 °C and 35 °C.

## 5. Conclusions

The organization and stability of the PI monolayer were influenced by the presence of P6, AA, and EAA in the subphase, as well as by temperature. The presence of P6 promoted a more flexible monolayer and increased its compressibility, likely through interactions with the PI headgroups. In turn, AA and EAA disrupted these interactions, leading to an increase in surface area per molecule, and hysteresis showed phase transitions that began at larger surface areas per molecule with increasing temperature. These results highlighted the potential of targeted interventions on membrane structure in the context of developing new antimicrobial drugs. EAA, as well as AA, may participate in hydrophobic interactions with both the monolayer and P6, which would enable better penetration of this components through lipid biological barriers, improvement in the stability and bioavailability in pharmaceutical formulations, and more effective delivery of active ingredients in the skin.

## Figures and Tables

**Table 1:** The composition of evaluated the systems.

Evaluated Systems	Composition [Number of Molecules]			
	Monolayer	Subphase		
	PI	AA	EAA	P6
PI *	1.20 × 10^16^	-	-	-
PI + AA *		2.30 × 10^16^	-	-
PI + EAA *		-	2.30 × 10^16^	
PI + P6		-	-	2.30 × 10^16^
PI + P6 + AA		2.30 × 10^16^	-	2.30 × 10^16^
PI + P6 + EAA		-	2.30 × 10^16^	2.30 × 10^16^

* Reference [16] informs on the former evaluation of monolayers 1–3, which were reassessed for comparison purposes.

**Table 2:** Characteristic parameters of π–A isotherms: A_lift-off_—surface area per molecule at the onset of surface pressure increase; A_collapse_—surface area per molecule at monolayer collapse; π_collapse_—collapse pressure (mN/m); χ (A_collapse_/A_lift-off_)—ratio of collapse area to lift-off area; a_LE/LC_—slope coefficient of the linear region of the π–A isotherm corresponding to the dynamic increase in surface pressure.

Evaluated Systems	Temperature (°C)	A_lift-off_ (Å^2^/molec.)	A_collapse_ (Å^2^/molec.)	π_collapse_ (mN/m)	χ	a_LE/LC_
PI	20	101.35	13.54	38.43	0.13	−0.571
	25	101.27	17.09	38.89	0.17	−0.594
	30	101.30	21.56	35.06	0.21	−0.573
	35	101.26	21.38	35.08	0.21	−0.572
PI + AA	20	101.53	24.11	37.96	0.24	−0.588
	25	102.80	33.73	21.23	0.33	−0.552
	30	101.54	18.23	27.67	0.18	−0.529
	35	101.54	34.00	32.41	0.33	−0.547
PI + EAA	20	100.72	13.50	41.74	0.13	−0.581
	25	101.10	22.05	38.46	0.22	−0.586
	30	101.86	33.27	35.11	0.33	−0.558
	35	101.60	13.68	29.97	0.13	−0.547
PI + P6	20	98.61	10.54	37.65	0.11	−0.554
	25	98.29	6.93	37.00	0.07	−0.551
	30	97.90	3.94	40.56	0.04	−0.554
	35	98.60	3.94	29.58	0.04	−0.512
PI + P6 + AA	20	101.68	10.19	29.16	0.10	−0.537
	25	100.76	12.44	24.44	0.12	−0.411
	30	101.59	12.36	29.76	0.12	−0.598
	35	101.83	11.21	30.79	0.11	−0.613
PI + P6 + EAA	20	100.79	11.67	41.50	0.12	−0.592
	25	101.67	11.87	30.97	0.12	−0.582
	30	101.54	11.41	31.34	0.11	−0.558
	35	101.55	11.04	30.35	0.11	−0.598

**Table 3:** Compressibility coefficient *C_S_*^−1^ [mN/m] values of the tested systems.

Acronym	Temperature			
	20 °C	25 °C	30 °C	35 °C
PI	46.23	48.18	48.22	49.95
PI + AA	49.39	51.58	47.53	44.60
PI + EAA	45.47	49.01	46.77	47.21
PI + P6	46.26	47.60	43.13	45.8
PI + P6 + AA	51.06	37.45	56,18	56.82
PI + P6 + EAA	48.35	53.82	48.11	54.82

**Table 4:** Isotherm compression reversibility coefficient *R*_v_ for loops 1–3 of PI monolayers with P6, AA, EAA peptides in the aqueous subphase.

Temperature			*R*_v_ (%) Parameter for the Evaluated Systems				
		PI	PI + AA	PI + EAA	PI + P6	PI + P6 + AA	PI + P6 + EAA
20 °C	loop 1	70.77	70.13	72.84	80.59	75.80	71.42
	loop 2	73.06	81.89	80.04	88.67	83.54	80.31
	loop 3	72.66	83.64	82.32	96.43	85.04	81.53
25 °C	loop 1	74.54	73.30	72.63	87.34	68.59	99.62
	loop 2	77.57	77.64	80.30	90.32	69.88	76.81
	loop 3	79.03	79.60	81.95	87.11	75.41	72.58
30 °C	loop 1	86.81	59.59	67.03	83.47	59.47	78.70
	loop 2	77.23	73.46	76.80	97.06	67.58	67.88
	loop 3	80.80	79.49	80.61	95.94	75.68	74.88
35 °C	loop 1	66.43	51.74	60.53	87.34	58.77	57.83
	loop 2	79.77	69.83	74.97	93.87	65.14	56.84
	loop 3	86.83	74.38	78.56	97.77	90.13	63.42

## Data Availability

The scientific data are available at Wroclaw Medical University, Department of Physical Chemistry and Biophysics.
